# Knowledge, Awareness, and Practice Related to Diabetic Foot Ulcer Among Healthcare Workers and Diabetic Patients and Their Relatives in Saudi Arabia: A Cross-Sectional Study

**DOI:** 10.7759/cureus.32221

**Published:** 2022-12-05

**Authors:** Sultan H Alsaigh, Raneem H Alzaghran, Dalal A Alahmari, Lama N Hameed, Kadi M Alfurayh, Khozama B Alaql

**Affiliations:** 1 General Surgery, King Fahad Specialist Hospital, Buraidah, SAU; 2 General Surgery, College of Medicine, Qassim University, Buriydah, SAU

**Keywords:** management of diabetic foot, foot ulcer, diabetic patients, healthcare workers, practice, awareness, knowledge, diabetic foot ulcer, dm

## Abstract

Background

Diabetes mellitus is a chronic progressive metabolic disorder characterized by high blood sugar affecting the whole body resulting in a significant impact on the quality of life for the patients and their families. Diabetes mellitus complications lead to morbidity, disability, and mortality and represent a serious global health issue threatening the health system worldwide and resulting in a critical economic impact for all countries, especially epidemic ones.

Objective

The objective of this study was to assess the level of knowledge and awareness regarding diabetic foot and related behaviour among diabetic patients and their relatives, as well as healthcare workers in Saudi Arabia.

Methods

A cross-sectional analytic study was conducted in 2022 on healthcare workers, diabetic patients, and their relatives above the age of 18 in Saudi Arabia by using a valid, pretested structured questionnaire. The collected data were analyzed using IBM SPSS Statistics for Windows, Version 26 (Released 2019; IBM Corp., Armonk, New York, United States).

Results

In this study, there was no correlation between the healthcare workers’ attitude and knowledge. A total of 131 healthcare workers were involved, and a majority of them had good knowledge regarding the predisposing factors of diabetic foot ulcers, and 63 (48.1%) had good knowledge regarding diabetes foot care. On the other hand, there was poor knowledge regarding the characteristics and complications of diabetes ulcers. This study showed various attitudes among healthcare workers regarding diabetic ulcer care. For example, they prioritized the prevention of ulcers over treatment (N=67, 51.1%), the majority of healthcare workers were very keen to wound care (N=77, 58.8%), and they believed that it was their responsibility to educate their patients about reducing re-ulceration (N=86; 65.7%). However, 52 participants (39.7%) considered management of diabetic foot ulcer time-consuming, 54 (41.2%) mentioned that if they had the opportunity, they would like to avoid taking care of the diabetic wound, and 51 (38.9%) reported non-satisfaction with diabetic wound care.

One hundred diabetic patients and 117 relatives also were involved, and only 41.3% of participants (patients and relatives) had good knowledge regarding diabetes mellitus. However, our findings also revealed that 91.65% of the participants had good knowledge and a favourable attitude towards diabetes mellitus and diabetic foot care. Nevertheless, even though the participants had good knowledge regarding foot care, they had poor practice, with 56.55% scoring poor on the assessment questionnaire.

Conclusion

Our study shows that most of the participants had good knowledge and attitudes but poor practices. This highlights the need for more efforts to educate the Saudi population about diabetes and its complications.

## Introduction

Diabetes Mellitus (DM) is a global health problem. It is a metabolic disorder characterized by high blood glucose levels due to either insulin hormone resistance or deficiency that can chronically affect the whole body [1]. The global prevalence of DM in 2019 among adults aged 20-79 years was about 463 million cases, which is estimated to rise to 578 million and up to 700 million by 2030 and 2045, respectively [2].

Saudi Arabia is considered the country with the second-highest number of diabetic patients in the Middle East and the seventh globally [3]. According to WHO reports in 2016, among the Saudi population, the prevalence of diabetes was 14.4% (13.8% among females and 14.7% among males). It was considered that is the cause of 5% of overall deaths in Saudi Arabia [4]. According to local studies, a cross-sectional study of the Alkharj region's population revealed a diabetes prevalence of 3.8% for females and 9.2% for males [5,6].

The complications of DM are classified into two major types. Macrovascular complications are long-standing damage to large vessels that lead to cardiovascular diseases, and microvascular complications are defined as damage to small vessels that lead to neuropathy, retinopathy, and kidney-related diseases [7]. On top of these complications is the nerve damage that results from diabetic neuropathy, which mostly damages the longer peripheral nerves that innervate the lower extremities, which affects 30% of DM-afflicted individuals, 50% of whom are over the age of 50, leading to a horrific increase in the risk of foot ulcers and lower-extremity amputation [8].

Diabetes foot is one of the most serious complications of diabetes. It is the primary cause of hospital admissions, amputations, and deaths in diabetic patients. The risk of death in the diabetic population with foot ulcers is 2.5 times higher than that of those without ulcers [9]. Diabetic foot complications result in a significant clinical and financial burden. The risk of complications increases with poor glycemic control, smoking, foot deformities, peripheral neuropathy, visual loss, and chronic kidney diseases [10].

Ischemic and neuropathic changes are the pathological basis of the development of diabetic foot ulcers. With improper foot care, ulcers are vulnerable to infection and therefore amputation. Almost 20% of moderate or severe diabetic foot infections result in amputations [11]. Proper foot care protects the feet of diabetic people. This includes examination of the skin, neurological and vascular assessments, choosing appropriate footwear, and trimming of toenails [10,12]. The care and management of diabetic foot have become an economic burden worldwide. In China, the total cost of care for diabetic foot ulcers for every patient has increased from ¥15,535.58 in 2014 to ¥42,040.60 in 2020 [13]. In addition to the economic burden, DM is a major cause of lower limb amputation globally. Another study in Canada showed that 81.8% of patients who underwent lower limb amputation were diabetics, and 93.8% of them had peripheral artery diseases [14].

Patient education plays a role in reducing the risk of diabetic foot ulcers and their complications [15]. Diabetic patients must be educated about foot care in order to decrease the risk of developing foot ulcers. This involves a good daily inspection of the feet and between the toes, daily feet washing and drying, and avoiding walking barefoot [16].

The involvement of a multidisciplinary team from different specialities in the management of diabetic foot reduces the risk of adverse outcomes and complications [17]. The proper care of diabetic foot ulcers should include good glycemic control, infection eradication, optimizing blood flow, and efficient wound care [18]. An important part of the management of diabetic foot ulcers is proper dressing, and there are different types of wound dressings. Surgical intervention is not always needed in the management of diabetic foot ulcers, but lower limb amputation could be a possible consequence in complicated cases. A study in Nigeria has identified the major predictors of lower limb amputation in patients with diabetic foot ulcers. These include an ulcer duration of more than one month, Wagner grade 4 or above, leukocytosis, proteinuria, osteomyelitis, and an infected wound [19].

Several studies examined diabetics' knowledge of foot care. Some of these studies showed that the patients had good knowledge but poor practice, which means that they lack education about diabetic foot [20-22]. Other studies showed that the patients themselves had adequate knowledge and attitude about a diabetic foot [23,24]. Furthermore, some studies found that healthcare providers in different countries have varying degrees of knowledge about diabetic feet, such as in Pakistan (40%) [25] and Sri Lanka (77.9%) [26]. A study conducted in Pakistan reported that the overall attitude of nurses towards patients with diabetic ulcers was positive [25]. However, increasing the level of practice is needed and this improvement could be achieved by providing courses to primary care physicians. Also, awareness programs for the early detection and care of diabetic foot problems in KSA.

The aim of our study was to measure how many healthcare workers, diabetic patients, and their relatives are aware of diabetes and diabetic foot ulcers in Saudi Arabia, and their behaviour related to it.

## Materials and methods

Study design, area, population, and sampling

This is a cross-sectional analytic study to assess diabetic foot knowledge, awareness, and related practice among diabetic patients, their relatives, and healthcare workers in a multicenter in Saudi Arabia. According to the Raosoft online sample size calculator (Raosoft Inc., Seattle, Washington, United States), the sample size was around 100 patients, 117 relatives, and 100 healthcare workers. All categories of healthcare workers (physicians, nurses, and physiotherapists), and all diabetic patients and their relatives above 18 years of age were included in the study. Participants who suffered from mental illness, were younger than 18 years, or did not answer the whole questionnaire were excluded.

Methods for data collection

After receiving written consent, the data was collected through a valid, pretested, structured, self-administered questionnaire developed using the help of others used in previous studies [23,25-27]. The data were collected by multiple-choice questions, Likert scale-related questions, and true or false type questions.

Statistical analysis

Descriptive statistics were summarized using numbers, percentages, and figures. The relationship between knowledge based on work-related surgical and medical areas was conducted using the Chi-square test. All value analyses were performed using IBM SPSS Statistics for Windows, Version 26 (Released 2019; IBM Corp., Armonk, New York, United States). P-value <0.05 was considered statistically significant. 

Ethical issues

We obtained approval for the study from the Qassim Regional Research Ethical Committee and adhered to ethics requirements (registration number: 1443-1167575). The research team obtained informed consent from all participants and followed all regulations while conducting this study.

## Results

Healthcare workers

Sociodemographic Characteristics

A total of 131 healthcare workers participated in this study and the majority were females (N=76, 58%) in comparison to males (N=55, 42%). The majority of the participants were from hospitals located in the central region of Saudi Arabia (64.9%) and the remaining were from hospitals in southern, northern, eastern, and western regions of Saudi Arabia. Of them, 62.6% were working in medicine-related departments and 37.4% were working in surgery-related departments. Of the participants, 50.4% were physicians, 42.7% were nurses, and 6.9% were physiotherapists. Most of the healthcare workers had a working experience of 6-10 years (45.8%) and ≤ 5 years (38.2%), and the majority of the participants (62.6%) had a wound care experience of ≤ 5 years (Table 1).

**Table 1:** Demographic characteristics of healthcare workers (N=131)

Variables		N (%)
Gender	Male	55 (42.0)
	Female	76 (58.0)
Workplace	Surgical-related	49 (37.4)
	Medicine-related	82 (62.6)
Hospital location	Central	85 (64.9)
	Eastern	11 (8.4)
	Southern	16 (12.2)
	Northern	4 (3.1)
	Western	15 (11.5)
Current profession	Physician	66 (50.4)
	Nurse	56 (42.7)
	Physiotherapist	9 (6.9)
Work experience	≤ 5 years	50 (38.2)
	6-10 years	60 (45.8)
	11-15 years	15 (11.5)
	16-20 years	3 (2.3)
	> 20 years	3 (2.3)
Wound care experience	≤ 5 years	82 (62.6)
	6-10 years	47 (35.9)
	11-15 years	1 (0.08)
	16-20 years	0 (0.0)
	> 20 years	1 (0.8)

Knowledge of Diabetic Foot

To assess the knowledge of the participants regarding diabetic foot ulcers, a questionnaire composed of 15 questions was used. The assessment was made based on work-related surgical areas such as general surgery, orthopedics, and wound care departments, among others. Any healthcare worker who worked in family medicine, cardiology, and endocrinology, or was a general physician, was considered a medical worker. Of the participants, 86.3% had good knowledge regarding the predisposing factors of diabetic foot ulcers. Furthermore, 48.1% had good knowledge about diabetic foot care with the majority of them (34.4%) being from medical specialities; 59.5% and 56.5% had poor knowledge concerning the characteristics and the complications of diabetic ulcers, respectively. We conducted a chi-square test to explore the relationship between knowledge questions and the workplace. A statistically significant association (p < 0.044) was found only with diabetic foot care questions (Table 2).

**Table 2:** Participants' knowledge of diabetic foot ulcers based on their work environment (N=131)

Variable		Work place		%	p-value
		Surgical-related (%)	Medical-related (%)		
Predisposing factor in ulcer formation	Good Knowledge	32.9	53.4	86.3	0.701
	Poor Knowledge	4.5	9.2	13.7	
Characteristics of ulcers	Good Knowledge	18.3	22.2	40.5	0.176
	Poor Knowledge	19.1	40.4	59.5	
Complications of ulcers	Good Knowledge	17.5	26	43.5	0.541
	Poor Knowledge	19.8	36.7	56.5	
Diabetic foot care	Good Knowledge	13.7	34.4	48.1	0.044
	Poor Knowledge	23.7	28.2	51.9	

Attitude Towards Diabetic Wound Care

This section was graded on a scale of 0 to 50, with 5 being the highest possible score for strongly agreeing and decreasing sequentially. The participants’ scores varied from 10 to 50 with a median of 26. Of the participants, 58.8% considered regular diabetic wound assessment necessary and 65.7% agreed that patient education regarding diabetic ulcers is their responsibility. On the contrary, 55.6% of the participants did not prefer to take care of diabetic wounds and 41% believed that diabetic ulcer treatment is more important than its prevention (Table 3).

**Table 3:** Results from the questionnaire regarding healthcare workers’ attitude towards diabetic foot care (N=131)

	Strongly Agree N(%)	Agree N(%)	Neither Agree nor Disagree N(%)	Disagree N(%)	Strongly Disagree N(%)
1. I think diabetic ulcer treatment is more important than ulcer prevention	25 (19.1)	29 (22.1)	10 (7.6)	21 (16.0)	46 (35.1)
2. I don’t think it is necessary to address diabetic wounds regularly	20 (15.3)	24 (18.3)	10 (7.6)	34 (26.0)	43 (32.8)
3. Diabetic wound care is time-consuming for me to carry out	20 (15.3)	32 (24.4)	30 (22.9)	22 (16.8)	27 (20.6)
4. In comparison with other job tasks, diabetic wound care is a low-priority task for me	22 (16.8)	12 (9.2)	32 (24.4)	33 (25.2)	32 (24.4)
5. If I have the opportunity, I would like to avoid taking care of diabetic wounds	25 (19.1)	29 (22.1)	31 (23.7)	2 2(16.8)	24 (18.3)
6. I don't have time to advise each patient individually on how to look after their wounds	21 (16.0)	10 (7.6)	14 (10.7)	52 (39.7)	34 (26.0)
7. It is not my responsibility to educate patients with diabetic ulcers on how to reduce re-ulceration	22 (16.8)	9 (6.9)	14 (10.7)	52 (39.7)	34 (26.0)
8. I cannot think about pain when cleaning diabetic wounds	21 (16.0)	24 (18.3)	34 (26.0)	30 (22.9)	22 (16.8)
9. I don’t like to care for diabetic wounds in my practice	24 (18.3)	49 (37.3)	16 (12.2)	22 (16.8)	20 (15.3)
10. I am not satisfied with caring for diabetic wounds	18 (13.7)	33 (25.2)	36 (27.5)	24 (18.3)	20 (15.3)

Diabetic patients and their relatives

Sociodemographic Characteristics

A total of 217 diabetic patients and their relatives were involved. One hundred and seventeen relatives were included in this study, with a majority of females (87.2%). Data were collected from the relatives of diabetic patients in the different regions of Saudi Arabia, primarily the central region (62.4%). The majority reported their parent/parents as the diabetic relation (78.6%) and only 17.1% reported their siblings as the diabetic relation (Table 4). A total of 100 diabetic patients were involved, mostly from the eastern region of Saudi Arabia (40%), with slightly more females (66%) and more than half (58%) being diabetic type 2. The majority of the diabetic patients were non-smokers (82%) (Table 4).

**Table 4:** Demographic characteristics of diabetic patients (N= 100) and their relatives (N=117)

Variables		Diabetic patients N (%)	Relatives N (%)
Gender	Male	34 (34)	15 (12.8)
	Female	66 (66)	102 (87.2)
Region of residence	Central	33 (33)	73(62.4)
	Eastern	40 (40)	6 (5.1)
	Southern	1 (1)	26 (22.2)
	Northern	3 (3)	3 (2.6)
	Western	23 (23)	9 (7.7)
Type of diabetes	Type I	42 (42)	58 (49.6)
	Type II	58 (58)	59 (50.4)
Relative relation	Mother/father	-	92 (78.6)
	Brother/sister	-	20 (17.1)
	Son/daughter	-	5 (4.3)
Duration since diagnosis	< 6 months	7 (7)	5 (4.3)
	> 6 months	23 (23)	23 (19.7)
	Since childhood	10 (10)	6 (51)
	> 5 years	60 (60)	83 (70.9)
Smoking	Yes	18 (18)	20 (17.1)
	No	82 (82)	106 (90.6)
Other chronic diseases	Dyslipidemia	16 (16)	16 (13.7)
	Hypertension	23 (23)	45 (38.5)
	Both	21 (21)	19 (16.2)
	None	40 (40)	37 (31.6)

Knowledge and Attitude

**Figure 1:**
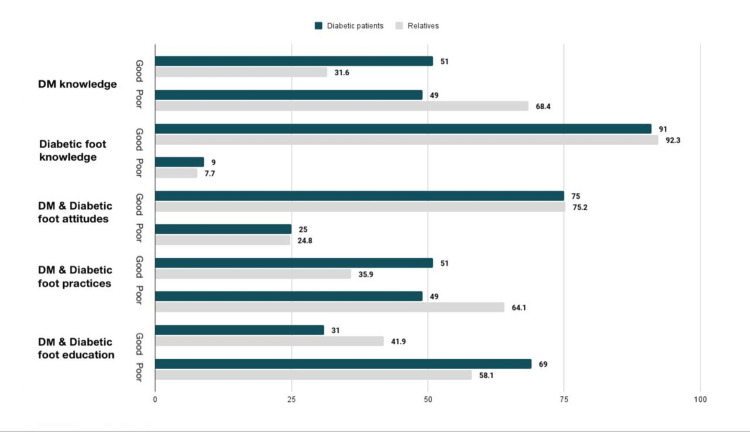
Knowledge, attitude, and practice of patients and relatives related to DM and diabetes foot DM: diabetes mellitus

For ease of comparison with other studies, each section’s scores were divided into two equal class intervals (poor and good) for both diabetic patients and their relatives. The scoring distribution for this sample according to these categories can be seen in (Figure 1). Concerning knowledge of DM, diabetic patients had better knowledge compared to their relatives by 19.4%. The majority of patients and relatives had good knowledge of diabetic foot and favourable attitudes towards DM and diabetic foot care. Interestingly, the results showed that despite this, a high percentage of the participants had poor education about DM and diabetic foot.

Diabetic patients’ education about DM and diabetic foot

**Figure 2:**
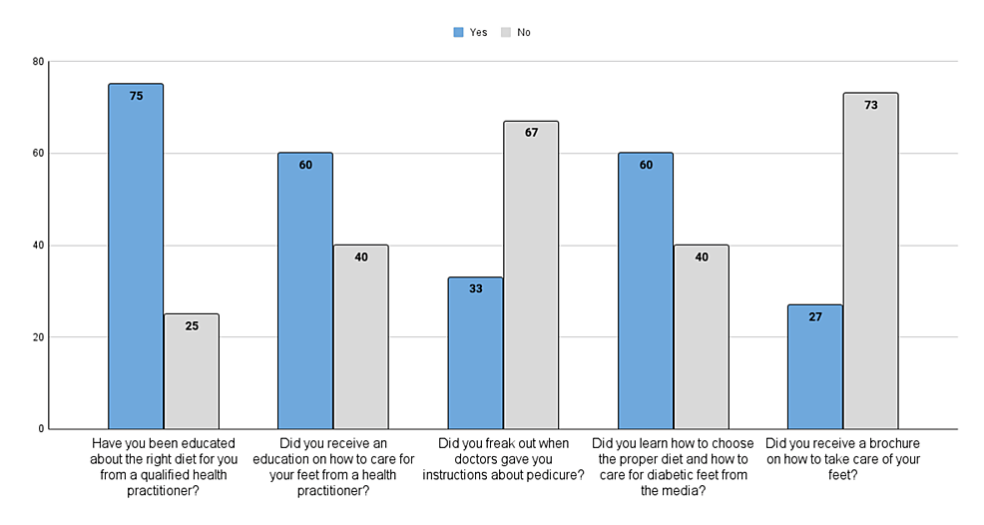
Source of education about DM and diabetic foot for diabetic patients DM: diabetes mellitus

A high proportion of patients (75%) reported positive education about diet from a qualified health practitioner while 60% learned how to choose the proper diet and care for diabetic foot from the media. Sixty percent reported receiving foot care education from a health practitioner, and only 27% had received brochures on diabetic foot care (Figure 2)

## Discussion

Most healthcare participants in our study were females (58%) and from central region hospitals in Saudi Arabia (64.9%). However, 62.6% of them were in the medicine-related department. Most healthcare workers were physicians (50.4%), and the duration of work experience of the majority (62.6%) was equal to or less than five years. The majority of healthcare workers in this study had good knowledge regarding the predisposing factors of diabetic foot ulcers (86.3%) and good knowledge about diabetic foot care (48.1%), which is helpful in patient care compared to a previous study conducted on nurses from Malaysia that found inadequate knowledge regarding diabetic foot ulcers and proper care for the diabetic foot [27]. However, that difference may be due to various healthcare workers' involvement in this study, as nurses were only 42.7% of total healthcare workers included in the study. Nevertheless, the knowledge regarding the characteristics and complications of diabetic ulcers in this study was poor. Therefore, it highlights how essential it is to raise this knowledge regarding diabetic ulcers for early detection and prevention of complications that enhance the patient’s life. There is a shortage of studies conducted on healthcare workers regarding diabetic foot ulcer knowledge and practice. We believe more studies to assess the knowledge can be helpful to detect the defects and fill the gaps. In addition, we believe that knowledgeable healthcare workers reflect early detection of diabetic ulcers, leading to a favourable prognosis.

This study was conducted among 100 diabetic patients and 117 relatives in Saudi Arabia. Similar to previous studies [28-30], the majority of the patients were females (66%) with 87.2% having type II diabetes. On the contrary, a recent study in Riyadh reported that the majority had type I diabetes [31]. The majority of patients were affected by diabetes for more than five years, and this will lead to many health impacts such as the increased risk of heart attacks, strokes, and kidney diseases [26]. This study shows that most diabetic patients do not smoke, similar to another study in which smokers represented only 6.25% of the sample [6]. Twenty-three percent of the patients have hypertension and 16% have dyslipidemia, while in another similar study, 61.4% have hypertension and 58.6% have dyslipidemia [24].

An individual's attitude is considered an essential characteristic that can influence their expectations [32]. For instance, a positive attitude can help prevent a disease from developing [33]. A number of studies have shown that healthcare workers have positive attitudes toward ulcer care [25-27]. Our study has a similar positive attitude regarding foot care. Table 3 showed the various attitudes of healthcare workers regarding diabetic ulcer care. A positive healthcare worker attitude is associated with a better disease outcome and fewer complications.

On the other hand, 39.7% of participants considered that diabetic foot ulcer management is time-consuming, 41.2% mentioned that If they have the opportunity they would like to avoid taking care of diabetic wounds, and 38.9% reported non-satisfaction with diabetic wound care. A previous study conducted in Saudi Arabia regarding nurses' attitudes demonstrated unsatisfactory scores and 10% of the nurses thought that preventing ulcers is time-consuming [34]. In this study, there was no correlation between the healthcare workers’ attitudes and knowledge. However, a study conducted on Belgian nurses revealed that a correlation between attitudes and knowledge was established. It also found that attitudes and work practices among healthcare workers correlated with one another [35].

Our study shows 41.3% of participants (patients and their relatives) have good knowledge regarding DM. The result is low in comparison to a similar study published in 2018, which showed the majority of patients (66.1%) had average diabetes knowledge and only 4.7% had high knowledge [36]. Therefore, increasing the patients' knowledge of DM and its complications should be taken into consideration in order to improve their outcomes. Our findings also revealed that 91.65% of the participants had good knowledge and a favourable attitude towards DM and diabetic foot care. These results are higher than a study done in 2017 in the Kingdom of Saudi Arabia, which found 55.2% of participants had high scores in knowledge concerning diabetic foot and future complications [21]. 

The majority of participants in this study got their information from the media and their healthcare providers. Even though the participants had good knowledge regarding foot care, they had poor practice with 56.55% scoring poor on the assessment questionnaire. Thus, greater care and effort need to be given to patients with DM.

Study limitations

The main limitation of our study is that the data was self-reported and may not accurately reflect reality. In addition, obtaining data from elderly patients was difficult as it was an electronic questionnaire.

## Conclusions

DM is a prevalent disease in Saudi Arabia. With poor glycemic control, many people suffer from its devastating complications. Our study shows that most of the participants have good knowledge and attitudes but poor practices. This highlights the need for more efforts to educate the Saudi population about diabetes and its complications.

## References

[1] Cho NH, Shaw JE, Karuranga S, Huang Y, da Rocha Fernandes JD, Ohlrogge AW, Malanda B (2018). IDF Diabetes Atlas: Global estimates of diabetes prevalence for 2017 and projections for 2045. Diabetes Res Clin Pract.

[2] Saeedi P, Petersohn I, Salpea P (2019). Global and regional diabetes prevalence estimates for 2019 and projections for 2030 and 2045: Results from the International Diabetes Federation Diabetes Atlas, 9(th) edition. Diabetes Res Clin Pract.

[3] Al Dawish MA, Robert AA, Braham R, Al Hayek AA, Al Saeed A, Ahmed RA, Al Sabaan FS (2016). Diabetes mellitus in Saudi Arabia: a review of the recent literature. Curr Diabetes Rev.

[4] (2019). Noncommunicable Disease Surveillance, Monitoring and Reporting: Diabetes country profiles. https://www.who.int/teams/noncommunicable-diseases/surveillance/data/diabetes-profiles.

[5] Aldossari KK, Aldiab A, Al-Zahrani JM (2018). Prevalence of prediabetes, diabetes, and its associated risk factors among males in Saudi Arabia: a population-based survey. J Diabetes Res.

[6] Al-Zahrani JM, Aldiab A, Aldossari KK (2019). Prevalence of prediabetes, diabetes and its predictors among females in Alkharj, Saudi Arabia: a cross-sectional study. Ann Glob Health.

[7] Zheng Y, Ley SH, Hu FB (2018). Global aetiology and epidemiology of type 2 diabetes mellitus and its complications. Nat Rev Endocrinol.

[8] Cole JB, Florez JC (2020). Genetics of diabetes mellitus and diabetes complications. Nat Rev Nephrol.

[9] Armstrong DG, Boulton AJ, Bus SA (2017). Diabetic foot ulcers and their recurrence. N Engl J Med.

[10] (2021). 11. Microvascular complications and foot care: standards of medical care in diabetes-2021. Diabetes Care.

[11] Edmonds M, Manu C, Vas P (2021). The current burden of diabetic foot disease. J Clin Orthop Trauma.

[12] Manickum P, Mashamba-Thompson T, Naidoo R, Ramklass S, Madiba T (2021). Knowledge and practice of diabetic foot care - a scoping review. Diabetes Metab Syndr.

[13] Lu Q, Wang J, Wei X, Wang G, Xu Y, Lu Z, Liu P (2020). Cost of diabetic foot ulcer management in China: a 7-year single-center retrospective review. Diabetes Metab Syndr Obes.

[14] Hussain MA, Al-Omran M, Salata K (2019). Population-based secular trends in lower-extremity amputation for diabetes and peripheral artery disease. CMAJ.

[15] Monami M, Zannoni S, Gaias M, Nreu B, Marchionni N, Mannucci E (2015). Effects of a short educational program for the prevention of foot ulcers in high-risk patients: a randomized controlled trial. Int J Endocrinol.

[16] Mishra SC, Chhatbar KC, Kashikar A, Mehndiratta A (2017). Diabetic foot. BMJ.

[17] Wang C, Mai L, Yang C (2016). Reducing major lower extremity amputations after the introduction of a multidisciplinary team in patient with diabetes foot ulcer. BMC Endocr Disord.

[18] Blakely M (2016). The use of best practice in the treatment of a complex diabetic foot ulcer: a case report. Healthcare (Basel).

[19] Ugwu E, Adeleye O, Gezawa I, Okpe I, Enamino M, Ezeani I (2019). Predictors of lower extremity amputation in patients with diabetic foot ulcer: findings from MEDFUN, a multi-center observational study. J Foot Ankle Res.

[20] Abdulghani HM, AlRajeh AS, AlSalman BH (2018). Prevalence of diabetic comorbidities and knowledge and practices of foot care among diabetic patients: a cross-sectional study. Diabetes Metab Syndr Obes.

[21] Algshanen MA, Almuhanna MF, Almuhanna AM (2017). Diabetic foot awareness among diabetic patients in Saudi Arabia. Egypt J Hosp Med.

[22] Mathers CD, Loncar D (2006). Projections of global mortality and burden of disease from 2002 to 2030. PLoS Med.

[23] Shamim M, Alhakbani MS, Alqahtani MS, Alharthi OS, Alhaqbani YJ (2021). Knowledge, attitude, and practice regarding diabetic foot care among Saudi and non-Saudi diabetic patients in Alkharj. J Family Med Prim Care.

[24] (2021). Wound Care Knowledge, Attitudes And Practice Among People With And Without Diabetes Presenting With Foot Ulcers In Guyana. https://www.woundsinternational.com/resources/details/wound-care-knowledge-attitudes-and-practice-among-people-and-without-diabetes-presenting-foot-ulcers-guyana.

[25] Bilal M, Haseeb A, Rehman A (2018). Knowledge, attitudes, and practices among nurses in Pakistan towards diabetic foot. Cureus.

[26] Kumarasinghe SA, Hettiarachchi P, Wasalathanthri S (2018). Nurses' knowledge on diabetic foot ulcer disease and their attitudes towards patients affected: A cross-sectional institution-based study. J Clin Nurs.

[27] Wui NB, Azhar AA, Azman MH, Sukri MS, Singh A, Singh H, Wahid AM (2020). Knowledge and attitude of nurses towards diabetic foot care in a secondary health care centre in Malaysia. Med J Malaysia.

[28] Alshammari ZJ, Alsaid LA, Parameaswari P, Alzahrani AA (2019). Attitude and knowledge about foot care among diabetic patients in Riyadh, Saudi Arabia. J Family Med Prim Care.

[29] Qadi MA, Al Zahrani HA (2011). Foot care knowledge and practice among diabetic patients attending primary health care centers in Jeddah city. JKAU Med Sci.

[30] Saber HJ, Daoud AS (2018). Knowledge and practice about the foot care and the prevalence of the neuropathy among a sample of type 2 diabetic patients in Erbil, Iraq. J Family Med Prim Care.

[31] AlOwais MM, Shido OA (2020). Knowledge and practice of foot care in patients with diabetes mellitus attending primary care center at Security Forces Hospital, Riyadh, Saudi Arabia: a cross-sectional study. J Family Med Prim Care.

[32] Petty RE (2019). Attitudes and Persuasion: Classic and Contemporary Approaches. https://www.taylorfrancis.com/books/mono/10.4324/9780429502156/attitudes-persuasion-richard-petty-john-cacioppo.

[33] Maylor M, Torrance C (1999). Pressure sore survey. Part 3: locus of control. J Wound Care.

[34] Kaddourah B, Abu-Shaheen AK, Al-Tannir M (2016). Knowledge and attitudes of health professionals towards pressure ulcers at a rehabilitation hospital: a cross-sectional study. BMC Nurs.

[35] Beeckman D, Defloor T, Schoonhoven L, Vanderwee K (2011). Knowledge and attitudes of nurses on pressure ulcer prevention: a cross-sectional multicenter study in Belgian hospitals. Worldviews Evid Based Nurs.

[36] Zowgar AM, Siddiqui MI, Alattas KM (2018). Level of diabetes knowledge among adult patients with diabetes using diabetes knowledge test. Saudi Med J.

